# Status of Nutrition in Hemodialysis Patients Survey (SNIPS): Nutrition Intake in Obese and Overweight vs. Healthy Weight Patients

**DOI:** 10.3390/life11020166

**Published:** 2021-02-21

**Authors:** Mona Boaz, Vered Kaufman-Shriqui, Odile Azoulay, Talia Weinstein

**Affiliations:** 1 Department of Nutrition Sciences, School of Health Sciences, Ariel University, Ariel 40700, Israel; veredks@ariel.ac.il; 2 Department of Nephrology, Rabin Medical Center, Beilinson Campus, Petah Tikvah 49100, Israel; odila@clalit.org.il; 3 Department of Nephrology, Tel Aviv-Sourasky Medical Center, Tel Aviv 62431, Israel; tweinste@tauex.tau.ac.il; 4 Department of Internal Medicine, Sackler Faculty of Medicine, Tel Aviv University, Tel Aviv 69978, Israel

**Keywords:** nutrition, obesity, overweight, hemodialysis

## Abstract

Elevated body mass index (BMI) has been associated with improved survival and fewer hospitalizations in hemodialysis patients; however, it is not clear that dietary intake is associated with increased BMI in hemodialysis patients. The present analysis was designed to compare energy and macronutrient intake and distribution, as well as compliance with the International Society of Renal Nutrition and Metabolism (ISRNM) dietary guidelines, by body weight status (overweight/obese vs. normal weight) in hemodialysis patients. The status of nutrition in hemodialysis patients survey (SNIPS) cohort is a cross-sectional study including a representative sample of individuals on hemodialysis treated in hospital dialysis centers throughout Israel. Of the 375 patients eligible for the current analysis, 60.1% had BMI ≥ 25 kg/m^2^ (overweight/obese). For each participant, the following measures were recorded: dietary intake, blood biochemistry, anthropometric and hemodynamic measures. These were compared by body weight status. Compared to their normal-weight counterparts, overweight/obese hemodialysis patients did not differ by energy and macronutrient intake, distribution of these nutrients in the diet. Regardless of body weight status, hemodialysis patients have poor compliance with ISRNM dietary guidelines.

## 1. Introduction

Overweight and obesity (OWOB) are common findings in hemodialysis patients [1,2]. The mean body mass index (BMI) in hemodialysis patients has increased over the last decades and is currently > 25 kg/m^2^ [3]. In the general population, OWOB is associated with excess energy consumption, which is stored as fat [4]. Increased adiposity is associated with a pro-inflammatory metabolic milieu, which greatly increases the risk of chronic diseases, including type 2 diabetes, hypertension, cardiovascular disease, and some cancers [5].

Compared to the general population, in which obesity is associated with increased morbidity and mortality [6], overweight/obese (OWOB) hemodialysis patients may have a survival advantage over their normal-weight counterparts [7,8]. This counterintuitive finding may reflect protection from protein-energy wasting, sequestration of uremic toxins in adipose tissue, and alterations in circulating cytokines. [9].

In contrast to the general population, a link between OWOB and dietary intake has not been established in hemodialysis patients. The present analysis aims to compare energy and macronutrient intake as well as distribution of these nutrients in the diet between OWOB and normal weight hemodialysis patients who participated in the Status of Nutrition In hemodialysis Patients Survey: SNIPS [10]. Additionally, the study was designed to compare the percentage of patients meeting ISRNM nutrition recommendations for the intake of energy, protein, sodium, and phosphorus [11] by body weight status.

## 2. Materials and Methods

### 2.1. Overall Study Design and Plan

The Status of Nutrition In hemodialysis Patients Survey: SNIPS was a multi-center, cross-sectional study designed to examine malnutrition risk in a large, representative sample of Israeli hemodialysis patients treated at hospital (rather than community) centers [10]. SNIPS estimated the intake of macronutrients, micronutrients, and fluids; the percentage of participants meeting ISRNM dietary recommendations for energy, protein, sodium, and phosphorus intake; biochemical, hemodynamic, and anthropometric measures [11].

### 2.2. Study Population 

In the framework of the SNIPS cohort, a representative sample of the Israeli hemodialysis population treated in hospital hemodialysis units (rather than community centers) was recruited. The patient population in each unit was stratified by age (in five-year categories), sex, ethnicity, years of dialysis (in five-year categories), and any diabetes (yes/no). Within each stratum, patients were assigned a number using an online number generator, and randomly selected for participation. The number of individuals from each stratum was proportionate to the target population at each center.

### 2.3. Inclusion and Exclusion Criteria

All patients randomly selected from each stratum were eligible for participation in SNIPS if they agreed to enrollment. However, patients with active malignancy and those receiving total parenteral nutrition or who were fed through a gastrostomy or jejunostomy tube were excluded from SNIPS participation.

### 2.4. Informed Consent

Patients randomly selected from each stratum and interested in SNIPS enrollment received a detailed, informed consent sheet explaining the study purpose, study procedures, possible benefits and potential harms of study participation, and potential knowledge gained from the endeavor. All participants provided signed informed consent prior to inclusion in the study. The study was approved by the Institutional Ethics Committee (Helsinki Committee) at each participating center and by the Israel Ministry of Health.

### 2.5. Dietary Intake

Dietary intake was assessed using a standard, multi-pass, 5-step 24-h recall, which employs a structured in-person interview recorded in writing by the investigator. In the first pass, the respondent reports all food consumed from midnight to midnight on the day prior to the interview. The second pass focuses on food intake between meals. Cooking method, portion size, and specific ingredients are queried in the third pass, expanding upon the information gathered during the first pass. The fourth pass is characterized by a review of the information gathered thus far and permitted the patient to make additions and/or corrections. In the fifth pass, the investigator uses a prepared list to probe the patient about frequently forgotten foods, including alcohol, beverages, snacks, and dietary supplements [12]. All dietary intake interviews were conducted by registered dietitians or physicians who had been trained in the above-mentioned 24-h recall method. The validity of this method for assessing dietary intake in hemodialysis patients has been long established [13]. Nutrition analysis of all 24-h recalls was performed by one registered dietitian. Dietary intake was analyzed using "Tzameret" Nutrition Analysis software (Israel Ministry of Health, Yirmiyahu St 39, Jerusalem, Israel https://www.health.gov.il/Subjects/FoodAndNutrition/Nutrition/professionals/Pages/Tzameret.aspx, accessed on 21 February 2021), which utilizes an Israeli nutrition database. Energy, macro-, and micronutrients were analyzed. Dietary intake for energy, protein, sodium, and phosphorus was dichotomized according to ISRNM guidelines for these nutrients as meeting or not meeting the stated recommendation.

### 2.6. Demographics, Medical History, Laboratory Values

Extracted from the patient electronic medical record were demographic and medical history data. Prescribed medications and nutrition supplements, blood chemistry, lipid profile, parathyroid hormone (PTH), complete blood count, and delivered dialysis dose (Kt/V) were recorded from the monthly medical evaluation proximal to the date of the 24-h diet recall.

### 2.7. Definitions

#### 2.7.1. Overweight and Obesity

Body weight was calculated as the mean of three post-dialysis measures, one on the day of the 24-h recall and the two post-dialysis measures immediately preceding it. Height was recorded to the nearest 0.5 cm. BMI was calculated as weight (kg)/height (m^2^). Ideal body weight (IBW) was defined 0.9 × H (cm)−88 in males and 0.9 × H (cm)−92 in females [14].

Individuals were categorized by BMI according to internationally accepted definitions: BMI 18.5 < 25 kg/m^2^ (healthy); BMI 25 < 30 kg/m^2^ (overweight), and BMI ≥ 30 kg/m^2^ (obese). Obesity and overweight were collapsed into a single group (OWOB), including all individuals with BMI ≥ 25 kg/m^2^ vs. not overweight/obese weight (BMI 18.5 < 25 kg/m^2^).

#### 2.7.2. Sample Size

The present report includes 375 individuals, omitting the three underweight individuals (BMI < 18.5 kg/m^2^) from the original SNIPS study sample of 378 participants. It was known that approximately 60% of the population was OWOB; thus, a between-group ratio of 3:1 was assumed. A sample size of 296 (222 OWOB) provided 80% power to detect a true, by-body size difference of not less than 150 ± 400 kcal.

#### 2.7.3. Data Analysis

All statistical analyses were performed on SPSS v. 25.0 (IBM Inc., Armonk, NY, USA). Categorical variables such as OWOB, sex, and comorbidities were described using frequency counts and are presented as n (%). Associations between categorical variables were assessed using the chi-square test. Continuous variables are described as mean ± standard deviation. Distributions of continuous variables were assessed for normality using the Kolmogorov-Smirnov test. The following variables had approximately normal distributions: delivered dialysis dose (Kt/V); creatinine; mean systolic blood pressure; blood calcium; the calcium-phosphorus product; and % of kcal provided by each of the macronutrients. All other continuous variables had distributions significantly deviating from normal. Continuous variables were compared by OWOB using the *t*-test for independent variables or the Mann-Whitney U or the as appropriate. All tests are two-sided and considered statistically significant at *p* < 0.05.

## 3. Results

Of the 375 SNIPS cohort members included in this analysis, 60.1% were OWOB. Of these, 41.7% were obese (BMI ≥ 30 kg/m^2^). BMI in the OWOB group was 30.6 ± 4.8 kg/m^2^, vs. 22.2 ± 1.6 kg/m^2^, *p* < 0.001.

Characteristics of the study population are presented by body weight status in Table 1. Differences in age, sex, years on hemodialysis, ethnicity (Jewish vs. other), family status, location of residence (home vs. long term care facility), difficulty with chewing/swallowing, hypertension, or cardiovascular disease did not differ by body weight status. Smoking was less frequently observed in OWOB individuals. Not surprisingly, diabetes was significantly more frequent comorbidity in people with vs. without OWOB. Fewer OWOB people required feeding assistance.

Table 2 presents blood and hemodynamic measures by body weight status. Blood glucose levels were significantly higher in the OWOB group, consistent with greater diabetes prevalence among these individuals. C-reactive protein and white blood cell count differed significantly by body weight status, and both were elevated in people with OWOB. High density lipoprotein cholesterol (HDL) was significantly lower in the OWOB group. Importantly, albumin, a measure of nutrition status, did not differ between people with vs. without OWOB and was lower than recommended values in both groups.

Presented in Table 3 are prescribed medications, compared by body weight status. Insulin was prescribed significantly more frequently to people with OWOB, consistent with the greater percentage of diabetes in this group; however, the percentage of people on oral glucose-lowering medications did not differ by group. Aspirin was also more frequently prescribed to the OWOB group, though the increase in CVD in this group was not significant.

Dietary intake is presented by body weight status in Table 4. Dietary intake did not differ by OWOB for any of the nutrients, nor was there a difference in the percentage of people prescribed oral nutrition supplements or intradialytic parenteral nutrition. This analysis was repeated comparing obese individuals (BMI ≥ 30 kg/m^2^) to non-obese, and again comparing intake across normal, overweight and obese categories. Statistically significant differences in dietary intake were not detected.

Table 5 presents the proportion of patients meeting the International Society for Renal Nutrition and Metabolism (ISRNM) dietary recommendations for people on hemodialysis by body weight status. Only a minority of people in either group (OWOB yes/no) met ISRNM dietary recommendations. The percentage of people meeting any of the ISRNM guidelines did not differ by body weight status. In another analysis, participants were categorized as obese (BMI ≥ 30 kg/m^2^) vs. not obese (BMI < 30 kg/m^2^), and compliance with ISRNM dietary recommendations were compared by body size. In this analysis, 34.3% of obese people (vs. 17.4% of non-obese) met the ISRNM recommendation for energy intake (*p* = 0.015). Additionally, 51.4% of obese vs. 26.8% of non-obese individuals met the ISRNM recommendations for protein intake, *p* = 0.002, the greatest level of compliance with ISRNM nutrition recommendations in the study population.

## 4. Discussion

The present study demonstrates that dietary intake did not significantly differ by body weight status. Further, regardless of body weight status, hemodialysis patients have poor compliance with ISRNM dietary guidelines.

The present study had 80% power to detect a difference of 150 ± 400 between OWOB and non-OWOB participants. The actual between-group difference was only 17 kcal. The study did not have adequate power to detect this difference, but the actual difference is so small as to be of no clinical relevance [15].

Only a minority of people in the present study met ISRNM guidelines for energy, protein, sodium, or phosphorus, regardless of body weight status. The greatest percentage of compliance was for protein among OWOB participants, but this did not significantly differ from the percentage of people without OWOB meeting this guideline (31.5%). Interestingly, a significantly greater percentage of obese vs. non-obese individuals met ISRNM recommendations for energy and protein intake. Serum albumin levels, which can reflect nutrition status and malnutrition risk [16], did not differ by OWOB and were below recommended levels irrespective of body weight status. Non-dietary factors that may lower serum albumin levels include inflammation, infection, and advanced age [17]. It is noteworthy that C-reactive protein levels, which were elevated in both groups, were nevertheless significantly greater in participants with OWOB, suggesting a more pronounced inflammatory state in these patients. The significantly elevated white blood cell (WBC) levels in patients with OWOB further suggest an underlying inflammatory state [18]. Indeed, a soluble form of extracellular toll-like receptor 4 (sTLR4), has been shown to be positively correlated with BMI and C-reactive protein, but negatively correlated with albumin and lean tissue index [19]. This may provide an underlying mechanism through which inflammation and malnutrition are associated in hemodialysis patients [20].

Consistent with findings in the general population, OWOB is associated with greater type 2 diabetes prevalence in hemodialysis patients [21]. One mechanism explaining the association between OWOB and type 2 diabetes is micronutrient deficiency; specifically, the nutrients thiamine [22], vitamin C [23], and B12 [24] have been implicated. However, by-body weight status differences in micronutrient intake were not observed in the present study. In fact, none of the nutrients measured differed significantly by body weight status.

Oral nutrition supplements are an efficient way to increase nutrition intake in patients who are not otherwise meeting their nutrition goals and are at risk for malnutrition [25]. Despite poor compliance with ISRNM nutrition guidelines, oral nutrition supplements were infrequently prescribed to people with or without OWOB. 

Limitations of the present study are related to its cross-sectional study design. This design precludes any discussion of causality because temporality cannot be ascertained. Thus, it is not possible to ascertain whether the few differences identified between people with vs. without OWOB are the result of, the cause of, or only associated with, body weight status. Additionally, the study population was a representative sample of people treated at hospital hemodialysis centers; individuals treated at community hemodialysis centers were not included. People treated at hospital dialysis centers in Israel tend to be older and have more comorbidities [26]. This may limit the generalizability of findings only to people treated in hospitals. On the other hand, hemodialysis centers in Israel, regardless of setting, are required to maintain a dedicated dietitian on staff, who reviews the nutrition status of patients each month, which suggests a similarity in nutrition care provided, whether the care is received in a hospital or in a community center. While quality assurance surveillance suggests a need to improve compliance with these treatment guidelines, compliance levels do not differ between hospital and community centers [27]. Another study limitation was the reliance on BMI to capture body composition. While clinically expedient, this measure does not express differences in muscle and fat mass that change with alterations in nutrition status. For example, ultrasound measures of muscle mass and subcutaneous fat were both reduced in hemodialysis patients compared to healthy controls [28]. Similarly, obese hemodialysis patients demonstrated reduced muscle and fat mass compared to population norms [29].

The present used a valid, well-accepted method to assess dietary intake in hemodialysis patients [13]. Further, the study had 80% power to detect a difference of at least 150 ± 400 kcal, based on the assumption that a difference smaller than this would be of little clinical consequence. By-OWOB differences in energy intake, intake of any other nutrient measured, or compliance with dietary guidelines were not observed. It is possible that this lack of difference reflects systematic under-reporting of intake by OWOB participants [30]. If the finding is not a function of systematic bias, and the nutrient intake of OWOB hemodialysis patients is truly not significantly different from hemodialysis patients without OWOB, then it would appear that OWOB in hemodialysis patients is associated with non-dietary factors, such as an inflammatory metabolic milieu. This notion is evidenced by the elevated C-reactive protein and WBC in the OWOB group.

In the future, prospective studies of body weight change together with contemporaneous measures of dietary intake and markers of inflammation would elucidate if OWOB precedes or results from inflammation, nutrition, or an interaction between the two.

## Figures and Tables

**Table 1:** Characteristics of the study population by body weight status: (OWOB yes/no).

Characteristic	OWOB N = 225	Not OWOB N = 150	*p*-Value
Age (years) *	63.7 ± 11.9	64.9 ± 14.5	0.78
**Sex (% female)**	47.6	44.4	0.72
Years of dialysis *	0.84 ± 2.9	2.2 ± 5.8	0.58
**Present smoking (%)**	3.6	13.0	0.04
**Jewish (%)**	61.9	70.4	0.31
**Family status**			0.85
**Married (%)**	72.5	66.7	
**Widowed (%)**	13.0	12.5	
**Divorced (%)**	8.7	12.5	
**Single (%)**	5.8	8.3	
**Resides at home (%)**	97.7	100	0.15
**Requires feeding assistance (%)**	1.2	9.3	0.03
**Difficulty chewing/swallowing (%)**	1.2	1.9	0.75
**Comorbidities**			
**Diabetes (%)**	57.1	27.8	0.001
**Hypertension (%)**	83.3	88.9	0.37
Cardiovascular disease (%) **	46.4	38.9	0.38

Data are presented as % of each group and compared by body weight status (OWOB: BMI ≥ 25 kg/m^2^ vs. no OWOB: BMI 25 < kg/m^2^) using the chi-square test. * Data presented as mean ± standard deviation. Distributions of continuous variables significantly deviated from normal, so were compared by OWOB using the Mann-Whitney U test. Nominal variables were compared by OWOB status using the chi-square test. ** Cardiovascular disease = history of one or more of the following indicated in the medical record: coronary heart disease (myocardial infarction, percutaneous coronary intervention, coronary artery bypass graft); stroke; peripheral vascular disease (intermittent claudication, amputation).

**Table 2:** Blood, hemodynamic and anthropometric measures by body weight status (OWOB yes/no).

Measure	OWOB N = 225	No OWOB N = 150	*p*-Value
**Dialysis dose delivered (Kt/V)**	1.38 ± 0.24	1.42 ± 0.25	0.65
Glucose (mg/dL) *	134.1 ± 61.9	101.9 ± 30.2	0.03
**Albumin (g/dL)**	3.7 ± 3.0	3.7 ± 3.4	0.49
**C-reactive protein (mg/dL)**	17.1 ± 33.7	6.7 ± 7.4	0.03
**Creatinine (mg/dL)**	7.4 ± 1.9	7.5 ± 1.8	0.84
**Urea (mg/dL)**	106.8 ± 47.2	105.3 ± 40.8	0.65
**Parathyroid hormone (pg/mL)**	379.5 ± 278.2	364.5 ± 430.4	0.08
**Calcium (mg/dL)**	8.4 ± 0.7	8.5 ± 0.9	0.14
**Phosphorus (mg/dL)**	5.2 ± 1.3	5.2 ± 1.8	0.77
**Calcium-Phosphorus product (mg^2^/dL^2^)**	44.2 ± 12.2	44.4 ± 14.9	0.74
**Hemoglobin (mg/L)**	10.8 ± 1.4	11.1 ± 1.5	0.34
**WBC (10^9^/L)**	7.0 ± 2.4	6.0 ± 1.9	0.02
**Lipid Profile**			
**Total cholesterol (mg/dL)**	160.6 ± 33.9	156.7 ± 43.6	0.34
**HDL (mg/dL)**	37.9 ± 10.9	48.3 ± 15.8	0.04
**LDL (mg/dL)**	86.2 ± 27.1	83.5 ± 41.7	0.36
**Triglycerides (mg/dL)**	145.7 ± 52.4	131.5 ± 71.3	0.19
**SBP (mmHg)**	135 ± 24	134 ± 24	0.98
**DBP (mmHg)**	68 ± 13	71 ± 13	0.20

Data are compared by body weight status (OWOB: BMI ≥ 25 kg/m^2^ vs. no OWOB: BMI < 25 kg/m^2^). Kt/V, creatinine, and mean SBP were normally distributed; thus they were compared by OWOB using the t-test for independent samples. All other continuous variables had distributions significantly deviating from normal, so they were compared by OWOB using the Mann-Whitney U test. Nominal variables were compared by OWOB using the chi-square test. WBC = white blood cell count; HDL = high density lipoprotein cholesterol; LDL = low density lipoprotein cholesterol; SBP = systolic blood pressure; DBP = diastolic blood pressure. * Glucose measures are not fasting values; rather, they were measured as part of the routine monthly blood chemistry evaluations performed on all hemodialysis patients.

**Table 3:** Medications and supplements prescribed to the study population by body weight status (OWOB yes/no).

	Percent of Population with Prescription in Medical Record		
Medication Prescribed	OWOB N = 225	No OWOB N = 150	*p*-Value
**Phosphate binders**	98.8	98.1	0.75
**Erythropoietin**	83.8	83.7	0.98
**Antihypertensive agents**	75.0	74.1	0.93
**Vitamin D (alpha D3)**	60.7	46.3	0.10
**Iron**	60.7	61.1	0.96
**Folic Acid**	67.9	72.2	0.59
B-vitamin Supplements *	78.6	75.9	0.73
**Aspirin**	60.7	35.2	0.003
**Statins**	51.2	37.0	0.13
**Oral anti-hyperglycemic agents**	7.1	3.7	0.31
**Insulin**	28.6	13.00	0.03
**Diuretics**	31.0	24.1	0.38

Data are presented as % of each group and compared by body weight status (OWOB: BMI ≥ 25 kg/m^2^ vs. no OWOB: BMI < 25 kg/m^2^) using the chi-square test. * Vitamin B supplements include one or more of the following: vitamin B1, B2, B3, B6, B12.

**Table 4:** Nutrition intake of study participants by body weight status (OWOB yes/no).

	OWOB N = 225	No OWOB N = 150	*p*-Value
**Energy (kcal/day)**	1433 ± 527	1450 ± 573	0.86
**Energy/kg IBW**	24 ± 10	24 ± 9	0.71
**Protein (g/day)**	68 ± 29	66 ± 25	0.69
**Protein (g/kg IBW)**	1.2 ± 0.6	1.1 ± 0.5	0.43
**Protein (% total kcal)**	19 ± 5	19 ± 4	0.77
**Fat (% total kcal)**	34 ± 11	35 ± 10	0.85
**Saturated fat (% total kcal)**	10 ± 5	11 ± 5	0.12
**Carbohydrates (% total kcal)**	47 ± 11	47 ± 14	0.89
**Fluids (mL/day)**	1104 ± 462	1111 ± 473	0.93
**Vitamin A (** **μg/day)**	525 ± 983	435 ± 437	0.53
**Thiamin (mg/day)**	0.6 ± 0.4	0.6 ± 0.3	0.99
**Riboflavin (mg/day)**	1.2 ± 0.6	1.2 ± 0.7	0.90
**Vitamin B6 (** **μg/day)**	1.1 ± 0.6	1.0 ± 0.5	0.10
**Vitamin B12 (** **μg/day)**	3.5 ± 6.8	2.7 ± 2.1	0.38
**Vitamin C (mg/day)**	67 ± 63	61 ± 73	0.68
**Vitamin E (mg/day)**	7 ± 6	6 ± 5	0.06
**Calcium (mg/d)**	361 ± 187	358 ± 225	0.94
**Phosphorus (mg/day)**	947 ± 714	874 ± 415	0.49
**Potassium (mg/day)**	1766 ± 890	1544 ± 862	0.15
**Sodium (mg/day)**	2291 ± 1049	2117 ± 989	0.33
**Iron (mg/day)**	7 ± 3	7 ± 4	0.54
**Oral Nutrition Supplements (%)**	11	13	0.69
**IDPN (%)**	0	6	0.11

Data are compared by body weight status (OWOB: BMI ≥ 25 kg/m^2^ vs. no OWOB: BMI 25 < kg/m^2^). IBW = Ideal Body Weight. IDPN = Intradialytic parenteral nutrition. With the exceptions of protein (% total kcal) and fat (% total kcal), all variables had distributions significantly deviating from normal, so they were compared by OWOB using the Mann-Whitney U test; protein (% total kcal) and fat (% total kcal) were compared by OWOB using the t-test for independent samples.

**Table 5:** The proportion of Patients Meeting International Society for Renal Nutrition and Metabolism Nutrition Recommendations for Hemodialysis Patients by body weight status (OWOB yes/no).

Nutrient (Intake per Day)	Recommended Intake Level	OWOB N = 225	No OWOB N = 150	*p*-Value
**Energy (kcal)**	30–35 kcal/kg/day *	28.6	27.8	0.92
**Protein**	1.2–1.4 g/kg/day *	41.7	31.5	0.23
**Sodium**	80–100 mmol/day *	21.4	18.5	0.68
**Phosphorus**	800–1000 mg/day plus binders if elevated *	23.8	16.7	0.32

Data are presented as % of each group and compared by body weight status (OWOB: BMI ≥ 25 kg/m^2^ vs. no OWOB: BMI 25 < kg/m^2^) using the chi-square test. * When a range of intake is presented, the lower cutoff was used to calculate the proportion of subjects meeting the requirement for that nutrient.

## Data Availability

Data is contained within the article.
